# Hepatotropic viruses as etiological agents of acute liver failure and related-outcomes among children in India: a retrospective hospital-based study

**DOI:** 10.1186/s13104-015-1353-z

**Published:** 2015-08-27

**Authors:** Anand Pandit, Leni Grace Mathew, Ashish Bavdekar, Shailesh Mehta, Gunasekaran Ramakrishnan, Sanjoy Datta, Yan Fang Liu

**Affiliations:** Department of Pediatrics and Neonatology, King Edward Memorial Hospital, Sardar Mudaliar Road, Rasta Peth, Pune, 411011 India; Christian Medical College, Vellore Child Health Unit 1, Vellore, 632004 India; GlaxoSmithKline Pharmaceuticals, Dr. Annie Besant Road, Worli, Mumbai, 400 030 India; GlaxoSmithKline Pharmaceuticals, #5 Embassy Links, SRT road, Bangalore, 560052 India; GlaxoSmithKline Vaccines, Rue Fleming 20, 1300 Wavre, Belgium; GlaxoSmithKline Vaccines, 150 Beach Road, #22-00 Gateway West, Singapore, 189720 Singapore

**Keywords:** Acute liver failure, Children, Etiology, Hepatitis A, India

## Abstract

**Background:**

Acute liver failure (ALF) is marked by a sudden loss of hepatic function and is associated with a high mortality rate in children. The etiology of ALF is shown to vary geographically. This study assessed the frequency of hepatotropic viruses as etiological agents of ALF in Indian children.

**Methods:**

This retrospective study enrolled children aged 0–18 years with confirmed ALF admitted to Christian Medical College, Vellore and King Edward Memorial Hospital and Research Center, Pune between January 2003 and December 2005. The frequency of hepatotropic viruses as etiological agents in children with ALF aged ≤18 years was calculated with 95 % confidence interval (CI). Descriptive analyses of demographic characteristics, clinical signs and symptoms of ALF, choice of treatment and outcomes were performed.

**Results:**

Of 76 children enrolled, 54 were included in the per-protocol analyses. Mean age of children with ALF was 5.43 years (standard deviation = 3.62); 51.9 % (28/54) were female. The percentage of children positive for anti-hepatitis A virus (HAV) IgM and hepatitis B surface antigen was 65.9 % (27/41; 95 % CI 49.4–79.9) and 15.9 % (7/44; 95 % CI 6.6–30.1), respectively. The final cause of ALF was HAV (36.3 %) followed by hepatitis B virus (HBV; 8.8 %). Before and during admission, encephalopathy was observed in 77.8 % (42/54) and 63.0 % (34/54) of children, respectively. A high number of children (46/54; 85.2 %) required intensive care and ALF was fatal in 24.1 % (13/54). The proportion of deaths due to HAV and HBV was 18.5 % (5/27) and 57.1 % (4/7), respectively.

**Conclusions:**

HAV and HBV were the most common etiological agents of ALF in Indian children. Primary prevention by vaccination against HAV and HBV in young children may be useful in the prevention of ALF due to viral hepatitis in India.

## Background

Acute liver failure (ALF) is a rare clinical syndrome marked by the sudden loss of hepatic function and a potentially fatal disease associated with a high mortality rate in children [1]. Worldwide, mortality associated with ALF is approximately 70 % without appropriate management or liver transplantation [2]. ALF is characterized by coagulopathy and hepatic encephalopathy that occurs within 8–26 weeks of onset of illness in patients without any underlying liver disease [3]. Early signs of illness include vomiting and poor feeding, while jaundice, irritability and reversal of sleep patterns may be variable during the early stages [2].

Currently, there are no specific therapies for ALF [1] except for orthotopic liver transplantation (OLT), which has improved survival rates from <20 % to approximately 60 % in ALF patients [4]. The causes of ALF include hepatotropic viruses, dengue, drug- and toxin-induced liver disease, and metabolic disorders [1, 5]. The etiology of ALF is shown to vary geographically where metabolic- and drug-induced conditions are common causes of ALF in Europe and North America; and hepatitis A virus (HAV) is one of the most common etiological agents in Asia and South America [5]. Infection with hepatotropic viruses such as HAV, hepatitis B virus (HBV) and hepatitis E virus (HEV) are suggested to be the most common causes of ALF in India [6]; however, the etiology of ALF in children differ from that in adults [5]. Therefore, the identification and awareness of etiological agents that cause pediatric ALF may facilitate and support preventative and treatment measures for childhood ALF in India. Although, a number of studies have been conducted to ascertain the etiology of ALF in different parts of India [7–13], the data obtained has been rather inconsistent. This study therefore assessed the frequency of hepatotropic viruses as etiological agents and outcomes of ALF in medical institutions located in two diverse parts of India—centers in southern and western India using for the first time a similar methodology at both centers.

## Methods

### Study design and participants

This retrospective, hospital-based study was conducted in Christian Medical College (CMC), Vellore, Southern India and King Edward Memorial (KEM) Hospital and Research Center, Pune, Western India. ALF cases between January 2003 and December 2005 were identified through the review of hospital discharge log-books using International Classification of Diseases (ICD)—9/10 codes from the admission/discharge database.

ALF was defined as cases with onset of encephalopathy ≤28 days after the onset of symptoms [6]. Diagnosis of ALF was based on clinical signs and symptoms and/or laboratory findings of liver failure [defined as International Normalized Ratio above the cut-off >2 [6] and increased bilirubin (total bilirubin exceeding the range of normal level as specified by the laboratory conducting the test.)] complicated by encephalopathy [or in the case of sub-acute liver failure (defined as cases with encephalopathy and/or ascites developing from 29 days up to 24 weeks/6 months after the onset of symptoms)] in patients without a previous history of liver disease.

Children aged 0–18 years with confirmed ALF admitted to the selected hospitals during the retrospective review period (between January 2003 and December 2005) were included in the study. Children were excluded from the study if medical records were unavailable for review or there was insufficient information to confirm the existence or classification of ALF. The study protocol and other related documents were reviewed and approved by the investigational center Institutional Review Board. The study was conducted according to Good Clinical Practice, the Declaration of Helsinki and the International Guidelines for Ethical Review of Epidemiological Studies. The study complied with applicable regulatory requirements. Since the data collected from the two study center databases were aggregated and de-identified in this study, no informed consent was required for the study.

### Laboratory assays

The results of liver function tests (peak prothrombin time, peak total bilirubin and peak direct bilirubin) and tests for viral hepatitis were collected from the medical records of children and analyzed. Test results for liver enzymes, alanine aminotransferase (ALT), aspartate aminotransferase (AST), total serum albumin and dengue fever serology were also reported descriptively.

### Data Analyses

For the determination of etiological agents of ALF, the per-protocol analyses included children identified with ALF in the hospital discharge log-book with no previous history of liver disease and whose medical records were available. The analysis for final causes of ALF was performed on all children diagnosed with ALF admitted to the selected hospitals during the study period. The number of ALF cases and the prevalence of etiological hepatotropic markers among ALF cases in children aged ≤18 years were reported with 95 % confidence interval (CI). Children with viral hepatitis, results of laboratory tests, choice of treatment and outcomes of ALF were reported. Results from the physical examination, present and past medical history were also detailed. All statistical analyses were performed using SAS version 9.1.3.

## Results

### Demography

A total of 76 children were enrolled into the study, of which 22 children were eliminated from the per-protocol analyses for protocol violation with respect to inclusion/exclusion criteria (n = 17) and for having an underlying medical condition not specified by the inclusion criteria (n = 5). Of the 54 children in the per-protocol analyses, 63 % (34/54) and 37 % (20/54) were from KEM hospital and CMC hospital, respectively. Mean age of children with ALF was 5.4 (standard deviation: 3.62) years; median age was 5.0 (range 0–18) years and 51.9 % (28/54) of children were female. All children were Indian.

### Etiological causes of viral hepatitis

**Fig. 1:**
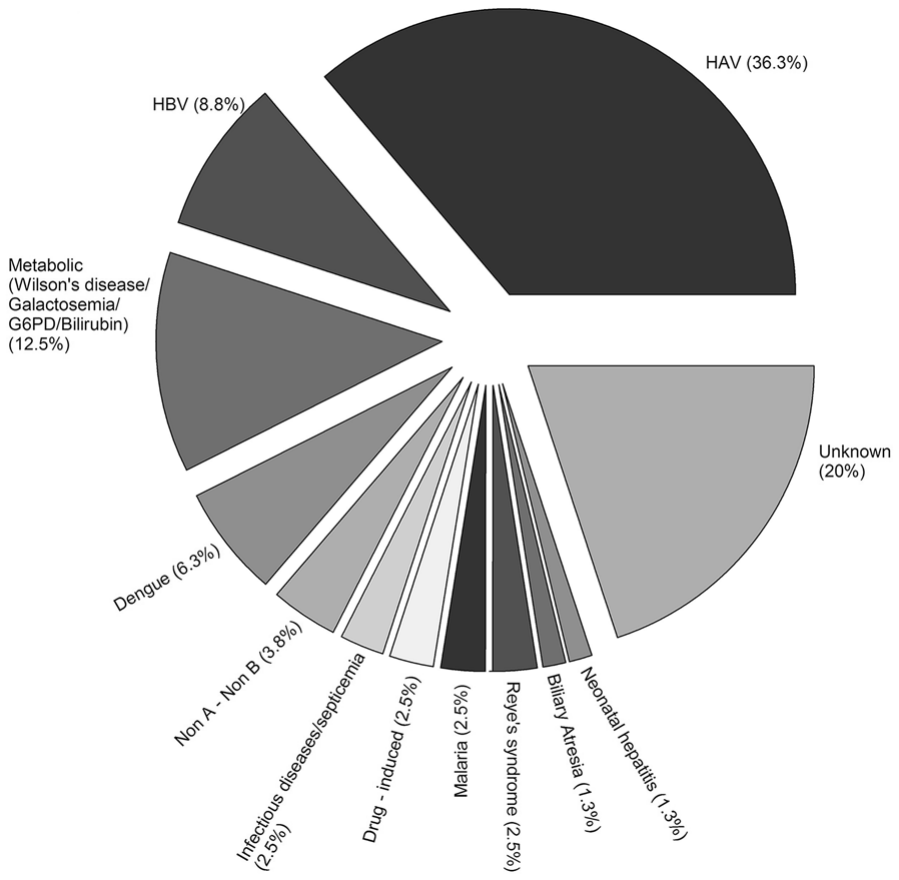
HAV, HBV and other causes of ALF (per-protocol) (N = 54). *HAV* hepatitis A virus; *HBsAg* hepatitis B surface antigen; *HBc* hepatitis B core antigen; *HCV* hepatitis C virus; *HEV* hepatitis E virus. Graphic program used: SigmaPlot 10.0

Among children who were tested, 65.9 and 15.9 % were positive for anti-HAV IgM and hepatitis B surface antigen (HBsAg), respectively. Two out of three tested children and one out of thirty tested children were positive for anti-hepatitis B core (anti-HBc IgM) and anti-HEV IgM antibodies, respectively. None of the eight tested children were positive for anti-hepatitis C virus (HCV) (Table 1). The final cause of ALF was HAV observed in 36.3 % of children followed by HBV observed in 8.8 % of children. Other causes of ALF are shown in Fig. 1.

**Table 1:** Presence of serological markers of viral hepatitis (per-protocol) (N = 54)

Characteristics	N^a^	n^b^	%^b^	95 % CI^c^
Anti-HAV^d^ IgM	41	27	65.9	(49.4, 79.9)
HBs-Ag^e^	44	7	15.9	(6.6, 30.1)
Anti-HBc^f^ IgM	3	2	66.7	(9.4, 99.2)
Anti-HCV^g^	8	0	0.0	(0.0, 36.9)
Anti-HEV^h^ IgM	30	1	3.3	(0.1, 17.2)

^a^Total number of children for whom the test was done

^b^number (percentage) of children for whom the test result was positive

^c^95 % confidence interval

^d^Hepatitis A virus

^e^Hepatitis B surface antigen

^f^Hepatitis B core antigen

^g^Hepatitis C virus

^h^Hepatitis E virus

### Assessment of liver function

The peak prothrombin time was above the normal limit in 74.1 % (40/54) of children; the peak total bilirubin and peak direct bilirubin was above the normal limit in 98.1 % (53/54) and 98.1 % (52/53) of children, respectively (Table 2). Peak ALT was above the normal limit for all 54 children tested. The total serum albumin levels were below the normal limit in 82.7 % (43/52) of children.

**Table 2:** Liver function tests (per-protocol) (N = 54)

Parameter	n (%)^a^	Above the normal upper limit^b^ n (%)^a^	Below the normal lower limit^c^ n (%)^a^
Alkaline phosphatase	20 (37.0)	4 (20.0)	–
Peak prothrombin time (s)	54 (100.0)	40 (74.1)	1 (1.9)
Peak total bilirubin (mg/ml^f^)	54 (100.0)	53 (98.1)	1 (1.9)
Peak direct bilirubin (mg/ml^f^)	53 (98.1)	52 (98.1)	1 (1.9)
Peak ALT^d^ (IU/L^g^)	54 (100.0)	54 (100.0)	–
Peak AST^e^ (IU/L^g^)	20 (37.0)	20 (100.0)	–
Total serum albumin (g/ml)	52 (96.3)	–	43 (82.7)

^a^Number (percentage) of children in each category

^b^Children for whom the test results were above the upper normal limit/value

^c^Children for whom the test results were below the lower normal limit/value

^d^Alanine aminotransferase

^e^Aspartate aminotransferase

^f^Milligram per milliliter

^g^International unit per milliliter

### Outcomes and treatment of ALF

Of 54 children, 85.2 % (n = 46) required intensive care. ALF was fatal in 24.1 % (13/54) of confirmed cases; 61.1 % (33/54) of children recovered and were discharged from the hospitals while 14.8 % (8/54) of children were discharged and their outcome was unknown. There were no reports of liver transplantation in any of the children.

The proportion of deaths attributed to HAV and HBV was reported to be 18.5 % (5/27; 95 % CI 6.3–38.1) and 57.1 % (4/7; 95 % CI 18.4–90.1), respectively.

### Medical history and physical examination

Jaundice was reported in 88.9 % (48/54) of children. One child had previous history of jaundice. Nearly all children (96.3 %; 52/54) had no exposure to industrial solvents or insecticides or mushroom poisoning and 90.7 % (49/54) of children did not have history of medication use in the last 3 months. Before hospital admission, 77.8 % (42/54) of children had encephalopathy of which 61.9 % (26/42) had grade 2 encephalopathy and 26.2 % (11/42) had grade 1 encephalopathy (Table 3). During admission, 63 % (34/54) of children had encephalopathy of which 47.1 % (16/34) had grade 5 encephalopathy (Table 3). A total of six children did not have encephalopathy before and after admission. The outcome of ALF cases related to the degree of encephalopathy is given in Table 4.

**Table 3:** Occurrence and severity of encephalopathy among children with ALF (per-protocol) (N = 54)

Presence of encephalopathy	Grade	n^a^	%^a^
Before admission			
Yes		42	77.8
	1^b^	11	26.2
	2^c^	26	61.9
	3^d^	4	9.5
	4^e^	1	2.4
	5^f^	0	0.0
	Missing	12	–
No		12	22.2
During admission			
Yes		34	63.0
	1	2	5.9
	2	9	26.5
	3	2	5.9
	4	3	8.8
	5	16	47.1
	Missing	2	–
No		20	37.0

^a^Number (percentage) of children with available results

^b^Child was confused and had mood changes

^c^Child was drowsy and displayed inappropriate behavior

^d^Child was stuporous but obeyed simple commands

^e^Child was comatose but was arousable by simple commands

^f^Child was in a deep coma and did not respond to any stimuli

**Table 4:** Outcome of ALF cases based on severity of encephalopathy (per-protocol) (N = 54)

Time point	Grade	Died (N = 13)^a^	Discharged with recovery (N = 33)^a^	Transferred out (N = 8)^a^	Total outcomes (N = 54)^a^
		n^b^	n^b^	n^b^	n^b^
Before admission					
Yes		11	25	6	42
	1^c^	2	7	2	11
	2^d^	8	16	2	26
	3^e^	1	1	2	4
	4^f^	–	1	–	1
	5^g^	–	–	–	–
	Missing	–	–	–	–
No		2	8	2	12
During admission					
Yes		13	15	6	34
	1	–	2	–	2
	2	–	8	1	9
	3	–	2	–	2
	4	–	2	1	3
	5	13	–	3	16
	Missing	–	1	1	2
No		–	18	2	20

^a^Total number of children in a given category

^b^Number of children with available results

^c^Child was confused and had mood changes

^d^Child was drowsy and displayed inappropriate behavior

^e^Child was stuporous but obeyed simple commands

^f^Child was comatose but was arousable by simple commands

^g^Child was in a deep coma and did not respond to any stimuli

Of 11 children tested for dengue fever serology, 45.5 % (5/11; 95 % CI 16.7–76.6) and 54.5 % (6/11; 95 % CI 23.4–83.3) tested positive for IgG and IgM, respectively.

## Discussion

This study assessed the etiology of ALF at one center each in Southern and Western India. The results from this study add to the data provided by notable Indian studies conducted previously [7–13]. In this study, HAV was the leading cause of ALF (36.3 %). A higher number of children were positive for anti-HAV IgM compared to other viral markers. This is similar to a previous study that showed a higher number of Indian children aged 0–15 years, positive for anti-HAV IgM versus other viral markers [9]. Previously, a study conducted in Argentina concluded that in the prevaccination period, HAV was the leading cause of fulminant hepatic failure and liver transplants in the pediatric population. Following the introduction of the vaccine, there was a striking decrease in both fulminant hepatic failure and liver transplants. There were no new cases reported since 2007 March and this was a large achievement decreasing burden of disease and costs associated with liver transplants [14].

HBV is widely reported as a common cause of ALF in adults from Asian countries [15]. However, HBV contributed only to 8.8 % of ALF cases in children in this study. This is consistent with a previous study that suggested HBV as a cause of ALF is less common in children than in adults [5].

ALF is a severe condition given that 85.2 % of children suffering from ALF required intensive care. Approximately, 24.1 % of confirmed ALF cases which proved to be fatal were lower than the global mortality rate (70 %) [2]. This finding may not reflect the true mortality rate attributed to ALF in India considering the children were referred to tertiary care, post-graduate hospitals where the specialized care, personnel and facilities available might have played a critical role in the survival of hospitalized children.

Etiology is the most important variable in predicting ALF outcome [6] and viruses such as HAV and HBV are good prognostic markers of outcomes. Another valuable predictor of outcome of ALF and the best indicator of survival is the peak prothrombin time [6, 11] which was observed in levels above the normal upper limit in 74.1 % of children. The total bilirubin peak, a useful prognostic indicator [10], was above the normal limit in 98.1 % of children in this study. Previous studies have shown that prolonged prothrombin time and elevated levels of total bilirubin above their respective normal upper limits were observed in patients who did not survive [10, 11]. Furthermore, it was reported that 63 % of children had encephalopathy. Severity of encephalopathy also help predict outcome of ALF cases [16], suggesting that the risk of death increases with progression through the grades of encephalopathy [17]. All children who died had encephalopathy at some point (before/during admission) in this study (Table 4).

Although treatment options such as OLT have improved survival rates of patients with ALF, the application is low as the treatment’s full potential has not been realized [18]. Determining the etiology and initial assessment of prognosis is suggested to provide useful information for ALF disease treatment and management [6]. HAV infection in early childhood is mostly asymptomatic or mildly symptomatic. Despite this acknowledgment, it is becoming increasingly evident that Indian children are susceptible to the disease in adolescence and adulthood [13], as seen in this study. Although the United States Advisory Committee on Immunization Practices has recommended universal HAV vaccination for children aged 12–23 months [19], vaccines against HAV in India are available only through private markets. Additionally, following the recognition of HBV as a cause of ALF, vaccination indicated against hepatitis B became a part of the national immunization program [20]. The national immunization programmes might start considering the inclusion of single-dose inactivated hepatitis A vaccines in their immunization schedules. This option seems to be comparable in terms of effectiveness, and is less expensive and easier to implement than the classical 2-dose schedule [21]. Moreover, since encephalopathy is a late feature of ALF and the severity of encephalopathy has a poor prognosis, preventative therapy in the way of early recognition and appropriate management is needed [5, 12]. Proper sanitary conditions and hygiene may help reduce the incidence of viral infection and timely diagnosis of infections due to hepatotropic viruses may help better manage the outcomes associated with ALF [12].

**Fig. 2:**
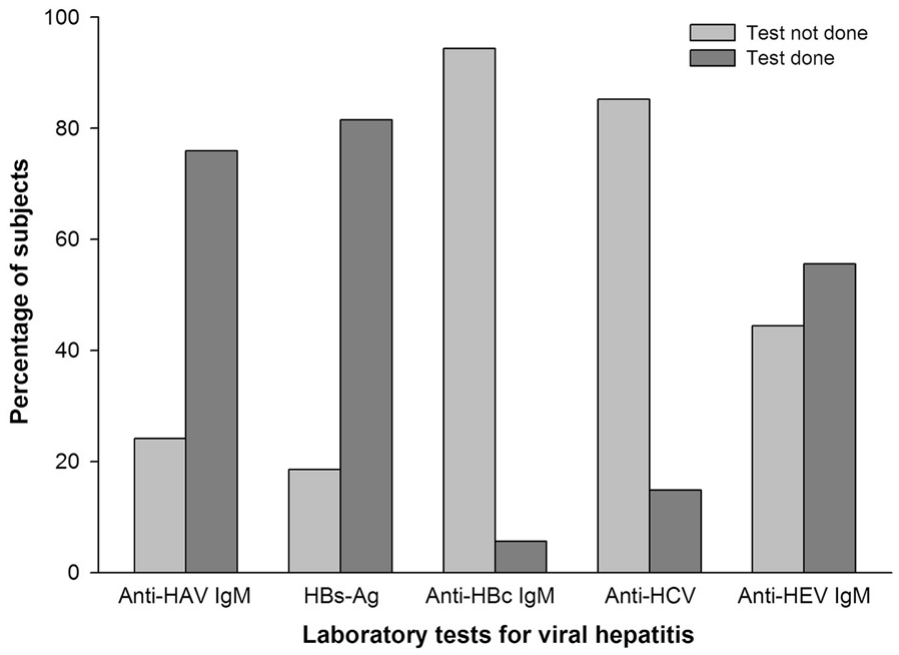
Laboratory tests for viral hepatitis (N = 80). 4 children had HAV and other etiological agents present. *HAV* hepatitis A virus; *HBV* hepatitis B virus. Graphic program used: SigmaPlot 10.0

The strength of our analysis is that all hospitalized children who were aged 0–18 years and reported ALF were included in this analysis. However, the findings of this study have to be interpreted in view of important limitations. First, the target sample size of 200 children was not achieved as one of the planned enrollment centers—Post Graduate Institute of Medical Education and Research (PGI), Chandigarh was closed before enrollment, and therefore, the study population may not be representative of the entire population in India. Second, the role of HAV and HBV as etiological agents of ALF may have been underreported. The reason being that the laboratory tests for viral markers were not performed for all children included in the analysis as these tests are not standard practice in India. Underreporting could also be due to limitations in the sensitivity of HEV IgM detection system as a marker of acute infection, especially in children. This could not be addressed due to the retrospective nature of the study (Fig. 2). The retrospective nature of the study also meant that viral genotypes of the patients, the viral load, the vaccination history and other such information not available in the medical records of the patients could not be studied.

## Conclusions

HAV was the leading cause of ALF among children in this study cohort. The results of this study suggest that a large proportion of ALF in this Indian study population are caused by vaccine-preventable infections such as hepatitis A and hepatitis B. Primary prevention by vaccination against hepatitis A and B viruses in young children may be useful in the prevention of ALF due to viral hepatitis in India.

## Abbreviations

ALF: acute liver failure
ALT: alanine aminotransferase
AST: aspartate aminotransferase
CI: confidence interval
CMC: Christian Medical College
HAV: hepatitis A virus
HBV: hepatitis B virus
HbsAG: hepatitis B surface antigen
HCV: hepatitis C virus
HEV: hepatitis E virus
ICD: International Classification of Diseases
KEM: King Edward Memorial Hospital and Research center
OLT: orthotopic liver transplantation
